# Time, the final frontier

**DOI:** 10.1002/1878-0261.70025

**Published:** 2025-03-24

**Authors:** Gautier Follain, Michal Dibus, Omkar Joshi, Guillaume Jacquemet

**Affiliations:** ^1^ Turku Bioscience Centre University of Turku and Åbo Akademi University Finland; ^2^ Faculty of Science and Engineering, Cell Biology Åbo Akademi University Finland; ^3^ InFLAMES Research Flagship Center University of Turku and Åbo Akademi University Finland

**Keywords:** cancer heterogeneity, circadian clock, live‐cell imaging, metastasis, temporal dynamics, temporal omics

## Abstract

Cancer's notorious heterogeneity poses significant challenges, as each tumor comprises a unique ecosystem. While single‐cell and spatial transcriptomics advancements have transformed our understanding of spatial diversity within tumors, the temporal dimension remains underexplored. Tumors are dynamic entities that continuously evolve and adapt, and relying solely on static snapshots obscures the intricate interplay between cancer cells and their microenvironment. Here, we advocate for integrating temporal dynamics into cancer research, emphasizing a fundamental shift from traditional endpoint experiments to data‐driven, continuous approaches. This integration involves, for instance, the development of advanced live imaging techniques, innovative temporal omics methodologies, and novel computational tools.

## Graphical abstract

This article advocates integrating temporal dynamics into cancer research. Rather than relying on static snapshots, researchers should increasingly consider adopting dynamic methods—such as live imaging, temporal omics, and liquid biopsies—to track how tumors evolve over time. These continuous approaches could reveal hidden patterns in cancer progression and metastasis, paving the way for more effective, personalized treatments.

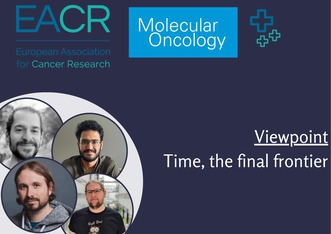

**Abbreviations**
CTCs: circulating tumor cells
ECM: extracellular matrix
EGFR: epidermal growth factor receptor

## 1 Introduction

**Fig. 1:**
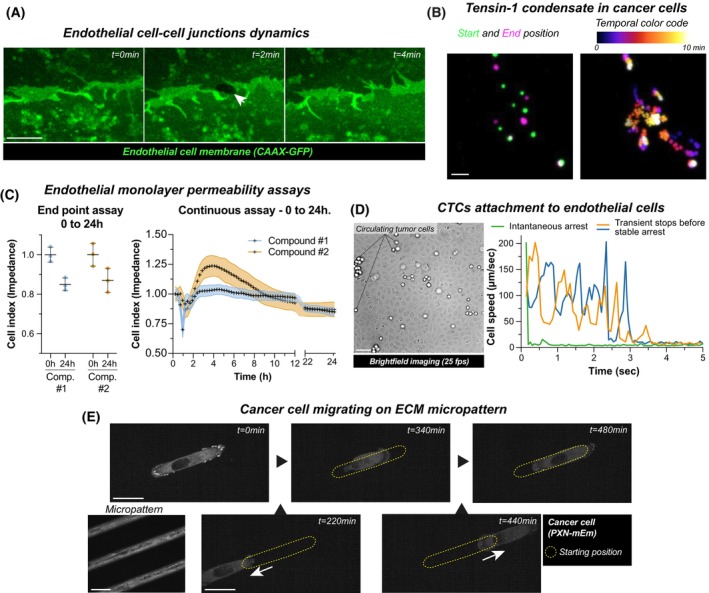
Time‐dependent measurements identify crucial biological phenomena. This figure highlights the importance of temporal resolution in capturing dynamic biological processes across various assays. (A) Dynamics of endothelial cell junctions: Live imaging using a Zeiss LSM880 Airyscan microscope (40×, water immersion) reveals that endothelial cell junctions—critical for blood vessel integrity—open and close rapidly. A single imaging plane is shown (scale bar: 3 μm). (B) Tensin‐1 condensates in cancer cells: Using spinning disk microscopy (63×, oil immersion), this panel tracks intracellular tensin‐1 (GFP‐tagged) in cancer cells over 10 min with images every 10 s. The left panel compares the first and last time points, while the right panel employs a temporal color code (Fire LUT) across all frames to highlight dynamic changes. A maximum intensity projection is provided (scale bar: 3 μm). (C) Endothelial monolayer permeability assays: Impedance measurements (monitor barrier function) were performed on a confluent endothelial monolayer after treatment. The left graph shows the start and end results (triplicates), while the right graph presents continuous measurements every 15 min for 24 h. Compounds 1 and 2 exhibit opposite effects and distinct kinetics, demonstrating the limitations of the endpoint assays. Error bars represent the standard deviation. (D) Circulating tumor cells attachment to endothelial cells: Live widefield imaging (20×; NA 0.45, Nikon CFI S Plan Fluor ELWD) of cancer cells perfused over endothelial monolayers in a microfluidic system. Cancer cells were individually tracked. The curves of the three selected cells illustrate different behaviors: green cells stably adhere at first contact, while orange and blue cells exhibit metastable adhesions before complete arrest. High temporal resolution is essential for studying these mechanisms. Scale bar: 50 μm. (E) Migrating cancer cells: Live imaging of a cancer cell expressing Paxillin‐mEmerald (PXN‐mEm) migrating on a micropatterned extracellular matrix track (100×; NA 1.4 oil, Zeiss Plan‐Apochromat). The top row shows selected time points of the cell's central position, while the bottom row depicts intermediate migration stages. An 8‐h recording with 20‐min intervals captures the cell's active exploration of its environment. Scale bars: 25 μm. (A–E) Data presented in this figure are for illustrative purposes only and are derived from one biological repeat.

Cancer's well‐known heterogeneity poses a significant challenge, as every tumor consists of a distinct ecosystem of cells and signals. Recent advancements in single‐cell and spatial transcriptomics and proteomics are revolutionizing our understanding of spatial diversity within tumors [1, 2]. However, time—a critical dimension—remains largely overlooked. Tumors are not static entities; they dynamically evolve and adapt throughout disease progression. Relying solely on static snapshots, such as endpoint measurements, obscures the dynamic interplay between cancer cells and their microenvironment (Fig. 1). This article advocates integrating more temporal dynamics into cancer research, illustrating how time‐dependent changes influence tumor behavior. By embracing both spatial and temporal facets, we can better understand and develop more effective strategies to interfere with cancer progression. The examples provided, while not exhaustive, demonstrate the potential of temporal analysis to uncover critical insights into cancer biology.

## 2 Cell signaling is heterogeneous in time

Dysregulated intracellular signaling is a central hallmark of cancer, driving hyperproliferation and other malignant behaviors. Traditionally, signaling has been viewed as a linear, static process, suggesting that once a pathway is activated, its activity remains uniformly elevated or suppressed. However, the dynamic nature of biological environments introduces temporal variability that shapes the evolution of complex signaling networks.

Many oncogenic pathways exhibit both short‐term and long‐term effects. Rapid signaling responses occurring within minutes—such as kinases modulating cytoskeletal organization or metabolic activity—are complemented by long‐term changes driven by gene transcription and protein synthesis. For example, epidermal growth factor receptor (EGFR) activation triggers immediate cytoplasmic events and initiates transcriptional programs that unfold over hours or days, ultimately influencing cell fate decisions [3].

Furthermore, critical cancer‐related pathways like NOTCH, TP53, and ERK demonstrate oscillatory or pulsatile activation patterns. These temporal ‘signatures’, characterized by both amplitude (signal strength) and frequency (rate of signal oscillation), are seldom captured by endpoint or population‐level experiments. For instance, assessing total ERK phosphorylation in a cell population at a single time point ignores that ERK activity can fluctuate within individual cells, leading to diverse cellular outcomes based on the timing and pattern of each activation pulse [4, 5, 6].

Monitoring signaling over extended periods is essential to fully understand these temporal dynamics. This will uncover how cells adapt or rewire their signaling networks in response to sustained or repeated stimuli (including oncogenes). Transient bursts of signaling activity may drive one set of cellular behaviors, while prolonged low‐level signaling could result in entirely different trajectories. By examining amplitude, frequency, and long‐term signaling response dynamics, we may be able to better understand how cancer cells acquire resistance, alter proliferation rates, or shift metabolic dependencies.

## 3 Metastasis is a dynamic process

Perhaps the best‐understood time‐dependent process in cancer biology is invasive cell migration. Metastasis—the leading cause of cancer‐related deaths—involves a sequence of events that enables tumor cells to detach from the primary tumor, invade new tissues, and survive in distant and diverse environments. Grasping the temporal nature of these processes is essential for unraveling how cancer cells reconfigure their intracellular machinery to migrate and disseminate.

A pivotal mechanism in metastasis is integrin‐mediated adhesion, which facilitates cancer cells to navigate through the stromal matrix during invasion [7]. Both integrin activation and their residence time at the plasma membrane fluctuate on the scale of minutes to hours, allowing cells to swiftly modulate their adhesion to the extracellular matrix (ECM). Modeling cell adhesion includes a time dependency linked to the receptor‐ligand contact time [8]. Indeed, the probability of establishing the molecular bond increases with time in proximity, as well as the reinforcement/multiplication of the bonds [9]. Integrin adhesion complexes recruit scaffold proteins, such as talin and vinculin over time that form robust anchoring points that must also be disassembled to permit forward movement. Concurrently, endosomal recycling redistributes adhesion receptors across the cell surface, enhancing the cell's ability to adapt to evolving ECM signals and leading to invasive migration [10]. Additionally, the ECM itself is continuously remodeled by cancer and stromal cells through secretion, proteolysis, and crosslinking, altering its composition and mechanical properties over extended periods [11].

Another example of temporal dynamics occurs when circulating tumor cells (CTCs) escape the bloodstream. Initially, CTCs form rapid but weak adhesions to transiently attach to vessel walls and resist the shear stress of blood flow. These brief interactions are succeeded by slower and stronger adhesions, which firmly anchor the cells and facilitate their exit from circulation to establish metastases [12, 13].

However, despite advancements in understanding the dynamic nature of cell migration and invasion, significant gaps remain in comprehending how these processes are regulated across different time scales. Rapid events, such as adhesion‐receptor engagement, must seamlessly integrate with slower processes like organelle and cytoskeletal reorganization, which in turn influence larger‐scale outcomes, such as cell migration. This hierarchical regulation poses a complex challenge, as the interplay between fast and slow processes dictates the overall behavior of migrating cancer cells.

## 4 Time influences the host

Cancer progression does not occur in isolation, as the host's physiological environment constantly changes. One critical example of time‐influenced host factors is the circadian clock, which plays a pivotal role in shaping cancer progression and response to treatment (e.g., cisplatin) [14].

The circadian clock is governed by a highly conserved set of core proteins, including BMAL1, CLOCK, PER, and CRY. These proteins orchestrate the temporal regulation of various physiological processes, maintaining homeostasis within the body. Recent findings have highlighted the prominent role of these clock genes and their associated signaling pathways across a wide spectrum of cancers, including breast, prostate, pancreatic, lung, colorectal, endometrial, and ovarian cancers [15].

Mechanistically, the circadian clock influences tumor growth through the cyclic variation of hormones and neurotransmitters, often causing perturbations that differ markedly from normal cellular behavior. For instance, disruptions in circadian gene expression in ovarian cancer have been shown to alter mitotic timing, contributing to uncontrolled cell proliferation [16]. Additionally, circadian rhythms play a crucial role in tumor dissemination. For instance, in breast cancer, the number of CTCs in the blood follows an oscillatory pattern, with peaks occurring during the rest phase. Notably, CTCs generated during the rest phase exhibit a higher propensity to metastasize, whereas those produced during the active phase lack metastatic capability [17]. The circadian clock also modulates the immune system, influencing the behavior of immune cells [18]. For example, an abnormal circadian clock was linked to T‐cell exhaustion and immune evasion [19].

Therefore, when conducting experiments in animal models, it is essential to consider how the host clocks (circadian, hormonal, aging, etc.) will influence the underlying processes.

## 5 Emphasizing the temporal dimension in cancer research

After exploring cancer's spatial complexities, it is now imperative to shift our focus toward the temporal dynamics that underpin cancer progression. However, generating, analyzing, and representing time‐resolved datasets presents significant challenges that must be addressed to fully harness the potential of temporal insights [20].

One primary strategy to capture dynamic data is through live‐cell imaging using innovative strategies [21]. Yet, despite its potential, fluorescence live‐cell imaging faces limitations, such as user bias and phototoxicity [22]. In particular, phototoxicity is often ignored if the sample does not die directly on the microscope. To overcome these hurdles, data‐driven microscopy acquisition presents an exciting way forward. These smart microscopy workflows leverage automated, real‐time data analysis to adjust imaging parameters dynamically, ensuring minimal perturbation while maximizing data acquisition [23]. These approaches hold the potential to record heterogeneous and critical phenomena as they unfold, facilitating the visualization of time‐dependent processes with unprecedented resolution and continuity.

Traditional transcriptomic methods often rely on pseudo‐time analyses to infer temporal sequences from static snapshots. However, emerging live‐omics approaches, such as Live‐seq [24], are revolutionizing the field by enabling real‐time transcriptomic recording of single cells. These techniques will allow us to track gene expression changes dynamically, providing a more accurate depiction of how cells respond and adapt over time.

Liquid biopsies can complement these methods by offering a minimally invasive means to monitor cancer progression over time. By analyzing CTCs, circulating tumor DNA, and extracellular vesicles from blood samples, liquid biopsies enable repeated sampling that captures the evolving genomic and proteomic landscape of tumors [25]. This dynamic approach could reveal emerging resistance mechanisms and metastasis‐related changes, providing critical insights into tumor evolution to inform timely therapeutic adjustments.

The vast and complex nature of time‐dependent data necessitates the development of robust analysis methods [20]. Deep learning algorithms and other advanced computational models are essential for deciphering patterns within these datasets. These tools can integrate data across multiple time scales, enabling the identification of unknown temporal interactions and predictive patterns that drive cancer progression [5].

Tackling the temporal dimension in cancer research signifies a fundamental shift from traditional endpoint experiments to data‐driven, continuous approaches. This paradigm shift is crucial for unraveling the intricate timing and spatial aspects of cancer progression. By advancing live imaging techniques, innovating temporal omics approaches, and harnessing computational tools, we can bridge the existing gaps in our temporal understanding of cancer biology.

## Conflict of interest

The authors declare no conflict of interest.

## Author contributions

GF and GJ wrote the manuscript. GF, MD, and OJ produced the experimental results in Fig. 1. All authors edited the manuscript.
